# An ensemble learning with active sampling to predict the prognosis of postoperative non-small cell lung cancer patients

**DOI:** 10.1186/s12911-022-01960-0

**Published:** 2022-09-19

**Authors:** Danqing Hu, Huanyao Zhang, Shaolei Li, Huilong Duan, Nan Wu, Xudong Lu

**Affiliations:** 1 College of Biomedical Engineering and Instrument Science, Zhejiang University, Hangzhou, China; 2 Key Laboratory for Biomedical Engineering, Ministry of Education, Hangzhou, China; 3 Department of Thoracic Surgery II, Peking University Cancer Hospital and Institute, Beijing, China

**Keywords:** Active sampling, Ensemble learning, Non-small cell lung cancer, Prognostic prediction

## Abstract

**Background:**

Lung cancer is the leading cause of cancer death worldwide. Prognostic prediction plays a vital role in the decision-making process for postoperative non-small cell lung cancer (NSCLC) patients. However, the high imbalance ratio of prognostic data limits the development of effective prognostic prediction models.

**Methods:**

In this study, we present a novel approach, namely ensemble learning with active sampling (ELAS), to tackle the imbalanced data problem in NSCLC prognostic prediction. ELAS first applies an active sampling mechanism to query the most informative samples to update the base classifier to give it a new perspective. This training process is repeated until no enough samples are queried. Next, an internal validation set is employed to evaluate the base classifiers, and the ones with the best performances are integrated as the ensemble model. Besides, we set up multiple initial training data seeds and internal validation sets to ensure the stability and generalization of the model.

**Results:**

We verified the effectiveness of the ELAS on a real clinical dataset containing 1848 postoperative NSCLC patients. Experimental results showed that the ELAS achieved the best averaged 0.736 AUROC value and 0.453 AUPRC value for 6 prognostic tasks and obtained significant improvements in comparison with the SVM, AdaBoost, Bagging, SMOTE and TomekLinks.

**Conclusions:**

We conclude that the ELAS can effectively alleviate the imbalanced data problem in NSCLC prognostic prediction and demonstrates good potential for future postoperative NSCLC prognostic prediction.

**Supplementary Information:**

The online version contains supplementary material available at 10.1186/s12911-022-01960-0.

## Background

Lung cancer is a type of cancer that begins in the lungs and may spread to lymph nodes or other organs in the body. It is the most diagnosed cancer and the leading cause of cancer death globally [1]. The two main types of lung cancer are small-cell lung cancer (SCLC) and non-small cell lung cancer (NSCLC). NSCLC is the most common type and accounts for about 85% of all lung cancer cases. The prognosis of NSCLC patients is poor and only 23.3% of cases can survive for more than 5 years [2].

In the era of precision medicine, more and more treatment options have become available. Besides the characteristics of cancer, cancer stage, treatment history, etc., prognosis is also of importance on the choice of complicated multidisciplinary treatment [3]. At present, surgery remains the only potentially curative modality for resectable NSCLC patients. However, cancer may recur at any time after surgery and seriously threaten the survival of postoperative patients [4]. Thus, it is critical to predict the prognosis of postoperative patients accurately to optimize the clinical decisions, such as adjuvant treatment selection and personalized follow-up plan, so that patients can receive proper management to improve the quality of life and even prolong the survival time [5, 6].

To accurately assess the prognosis of patients, researchers have adopted multiple machine learning algorithms to develop prognostic models by exploiting various data like clinical, imaging, and genomic data [7]. Although these models are capable of mining nontrivial knowledge from historical data [8–12], the imbalanced data problem is still a bottleneck of building a robust prognostic prediction model, especially for patients who relapsed or died shortly after surgeries, which causes the algorithms to bias the majority-class cases and affects the predictive performance [13]. Therefore, we need an effective strategy to counteract this problem.

In this paper, we propose a novel approach, i.e., ensemble learning with active sampling (ELAS), to alleviate the problem caused by imbalanced data. Active sampling has shown great potential to deal with the imbalanced data problem [14–17]. ELAS develops the first base classifier using a balanced initial training data seed, and then applies the active sampling mechanism to query samples to update the base classifier. Next, the base classifiers that achieve good performances on an internal validation set are integrated as the ensemble model. To evaluate the proposed method, extensive experiments were conducted on a clinical dataset consisting of 1848 postoperative NSCLC patients collected from a Chinese Cancer Hospital. Experimental results indicate that the ELAS outperforms several benchmark models, which shows the ability to alleviate the imbalanced data problem in postoperative NSCLC prognostic prediction.

## Methods

**Fig. 1:**
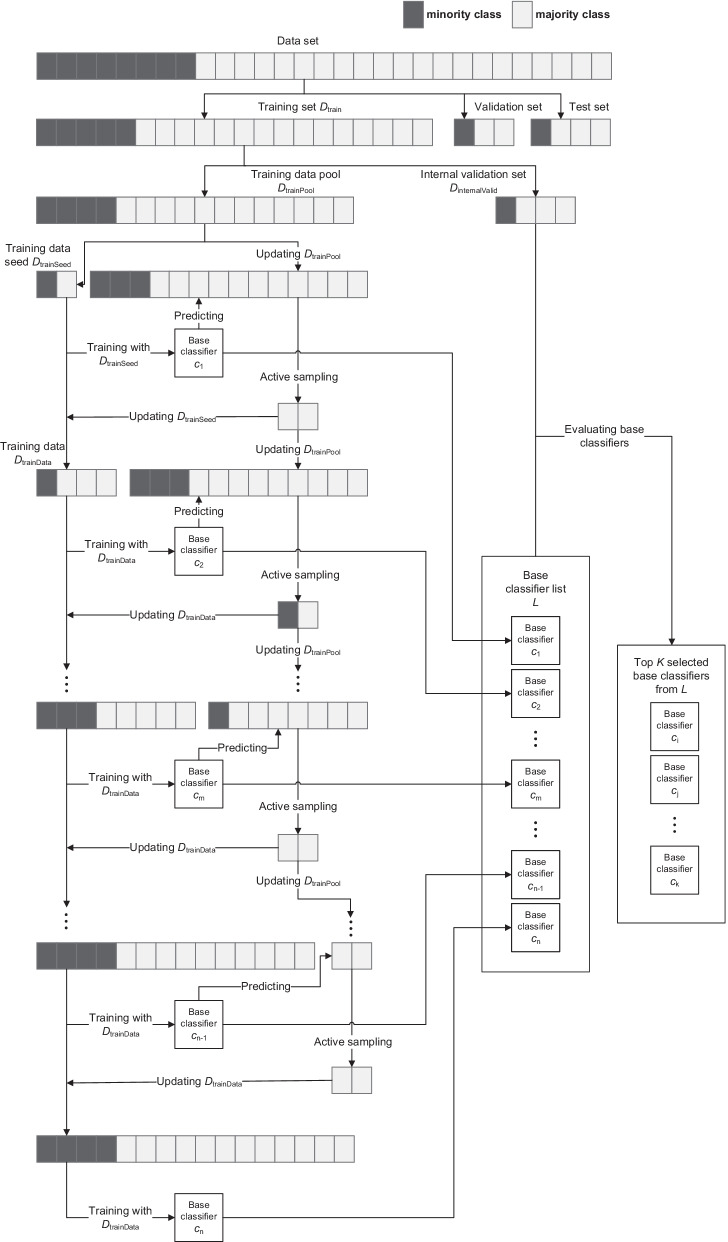
The process of the ELAS

Prognostic prediction for postoperative NSCLC patients is a typical imbalanced learning problem, especially for short-term prognosis prediction. Therefore, directly applying the traditional machine learning algorithms may lead to poor performance [13]. In this study, we propose the ELAS to alleviate the problem. Figure 1 illustrates the process of ELAS. The ELAS mainly consists of three parts, i.e., data initialization, active sampling, and model ensemble. We will elaborate on the details of the ELAS as follows.

### Data initialization

For training set $D_{{{\text{train}}}} = \{ x_{1} ,x_{2} , \ldots ,x_{{N_{{{\text{train}}}} }} \}$ where *x* is the patient sample and $N_{{{\text{train}}}}$ is the sample size of the training set. Before active sampling, we first randomly select 20% of the samples from the $D_{{{\text{train}}}}$ as the internal validation set $D_{{{\text{internalVal}}}}$. Note that the $D_{{{\text{internalVal}}}}$ is designed for the selection of the base classifiers in the ELAS model, which is different from the traditional validation set $D_{{{\text{val}}}}$ for hyperparameter selection or early stopping. And the remaining 80% of samples in $D_{{{\text{train}}}}$ are regarded as the training data pool $D_{{{\text{trainPool}}}}$ with sample size $N_{{{\text{trainPool}}}}$. When obtaining the $D_{{{\text{trainPool}}}}$, we randomly select $N_{{{\text{seed}}}} /2$ samples with no replacement from the majority class and minority class of $D_{{{\text{trainPool}}}}$ respectively as a balanced initial training data seed $D_{{{\text{trainSeed}}}}$ to train the first base classifier, where $N_{{{\text{seed}}}}$ is the sample size of the $D_{{{\text{trainSeed}}}}$. And the $D_{{{\text{trainPool}}}}$ is updated by removing the samples in the $D_{{{\text{trainSeed}}}}$.

### Active sampling

Using the balanced $D_{{{\text{trainSeed}}}}$, we train the first base classifier $c_{1}$ with any reasonable supervised machine learning algorithms. When the first base classifier $c_{1}$ is trained, we employ it to predict the risks of samples in the $D_{{{\text{trainPool}}}}$ and select the $N_{{{\text{batch}}}}$ most informative samples from $D_{{{\text{trainPool}}}}$ using any reasonable query strategies. In this study, we employ the ranked batch-mode sampling (RBMS) described in the literature [18] as the query strategy. In comparison with the traditional active learning query strategies like uncertainty sampling, RBMS uses Eq. (1) to assign the final scores for a batch of samples not only considering the informativeness of each sample but also the similarity between the samples and the already selected ones.

1 $$\begin{array}{*{20}c} {S_{{{\text{final}}}} = \alpha \times \left( {1.0 - S_{{{\text{similarity}}}} } \right) + (1.0 - \alpha ) \times S_{{{\text{uncertainty}}}} } \\ \end{array}$$

Note that the *α* parameter is responsible for weighting the impact of similarity score $S_{{{\text{similarity}}}}$ and uncertainty score $S_{{{\text{uncertainty}}}}$ in the sample’s final score $S_{{{\text{final}}}}$. Using Eq. (2), *α* leads the query strategy to prioritize diversity on the initial iterations where the ${\text{N}}_{{{\text{trainData}}}}$ is much smaller than the $N_{{{\text{trainPool}}}}$ while, with the increase of the queried samples, shift the priority to samples in which the classifier is uncertain about. $N_{{{\text{trainData}}}}$ is equal to $N_{{{\text{seed}}}}$ at the first active sampling iteration.

2 $$\begin{array}{*{20}c} {\alpha = \frac{{N_{{{\text{trainData}}}} }}{{N_{{{\text{trainPool}}}} + N_{{{\text{trainData}}}} }}} \\ \end{array}$$

To determine the uncertainty of the sample, the RBMS uses the least confident uncertainty score. Let $y_{{x_{i} }}^{j}$ be the probability of a sample $x_{i}$ belonging to class *j* predicted by the classifier, then the uncertainty score can be calculated by Eq. (3).

3 $$\begin{array}{*{20}c} {S_{{{\text{uncertainty}}}} = 1.0 - \mathop {\max }\limits_{j} y_{{x_{i} }}^{j} } \\ \end{array}$$

Moreover, RBMS employs Eq. (4) to find the similarity score, where $x_{i}$ is the current sample, $D_{{{\text{estimated}}}}$ is the dataset including samples in $D_{{{\text{trainData}}}}$ and the selected samples in this query round. $\emptyset$ is the similarity function to measure the distance between the $x_{i}$ and the sample in $D_{{{\text{estimated}}}}$. We used the Euclidean distance as the similarity function in this study.

4 $$\begin{array}{*{20}c} {S_{{{\text{similarity}}}} = \mathop {\max }\limits_{{x_{j} \in D_{{{\text{estimated}}}} }} \emptyset \left( {x_{i} , x_{j} } \right)} \\ \end{array}$$

Based on the RBMS, we can avoid the sub-optimal sample selection caused by traditional active learning query strategies when selecting $N_{{{\text{batch}}}}$ informative samples. The queried $N_{{{\text{batch}}}}$ patient samples are added into $D_{{{\text{trainSeed}}}}$ as the new training data $D_{{{\text{trainData}}}}$ and removed from $D_{{{\text{trainPool}}}}$. So far, the first active sampling process is done, and we obtain the first classifier $c_{1}$, new training data $D_{{{\text{trainData}}}}$, and training data pool $D_{{{\text{trainPool}}}}$. Based on the new $D_{{{\text{trainData}}}}$ and $D_{{{\text{trainPool}}}}$, we can start the next round of active sampling process until not enough samples in $D_{{{\text{trainPool}}}}$ can be sampled into $D_{{{\text{trainData}}}}$ for base classifier development. During each active sampling iteration, one base classifier is trained and used to query new samples for the next base classifier. All the trained base classifiers during this process are stored in the base classifier list *L* waiting for the final base classifier selection. In this study, we do not use the stop criteria to early terminate the training process [19–21], because the discrimination ability of the base classifier does not always improve with the addition of queried samples when using the real clinical data.

### Model ensemble

After the active sampling, we can obtain a base classifier list *L* with $\frac{{N_{{{\text{trainPool}}}} - N_{{{\text{seed}}}} }}{{N_{{{\text{batch}}}} }} + 1$ base classifiers, where $N_{{{\text{trainPool}}}}$ is the sample size of the $D_{{{\text{trainPool}}}}$ before training data seed sampling. Among these base classifiers, we select top *K* base classifiers with the best prediction performances on the internal validation set $D_{{{\text{internalVal}}}}$ for the ensemble model.

However, the $D_{{{\text{internalVal}}}}$ only accounts for 20% of the $D_{{{\text{train}}}}$, which may lead the selected base classifiers to overfit this $D_{{{\text{internalVal}}}}$ and deteriorate the generalization ability of the ensemble model. Thus, we apply a stratified fivefold cross-validation mechanism to generate the $D_{{{\text{internalVal}}}}$. Each fold is regarded as one $D_{{{\text{internalVal}}}}$ for base classifier evaluation, and the remaining 4 folds are combined as the $D_{{{\text{trainPool}}}}$ for base classifier training. Using this strategy, each sample in the $D_{{{\text{train}}}}$ will be used to evaluate and select base classifiers, and we can obtain 5 base classifier lists where each list corresponds to a $D_{{{\text{trainPool}}}}$ to avoid overfitting to one specific $D_{{{\text{trainPool}}}}$.

Moreover, we also notice that the different initial training data seed $D_{{{\text{trainSeed}}}}$ will lead to the different first base classifier and the following active sampling results and then the different subsequent base classifiers. To obtain more stable and robust prognostic prediction performance, we initialize $D_{{{\text{trainSeed}}}}$ $T_{{{\text{seed}}}}$ times with different random seeds and repeat the whole active sampling process separately to obtain $T_{{{\text{seed}}}}$ base classifier lists during each $D_{{{\text{internalVal}}}}$ fold. Thus, when using fivefold cross-validation for multiple $D_{{{\text{internalVal}}}}$ generations and $T_{{{\text{seed}}}}$ times $D_{{{\text{trainSeed}}}}$ initializations, we can obtain a total of $5 \times T_{{{\text{seed}}}}$ base classifier lists. We select the top K base classifiers from each *L* based on their performances on corresponding internal validation sets. The ELAS will average the $5 \times T_{{{\text{seed}}}} \times K$ base classifiers’ outputs as the final ensemble result. The details of the whole training process of the ELAS are given in Algorithm I.
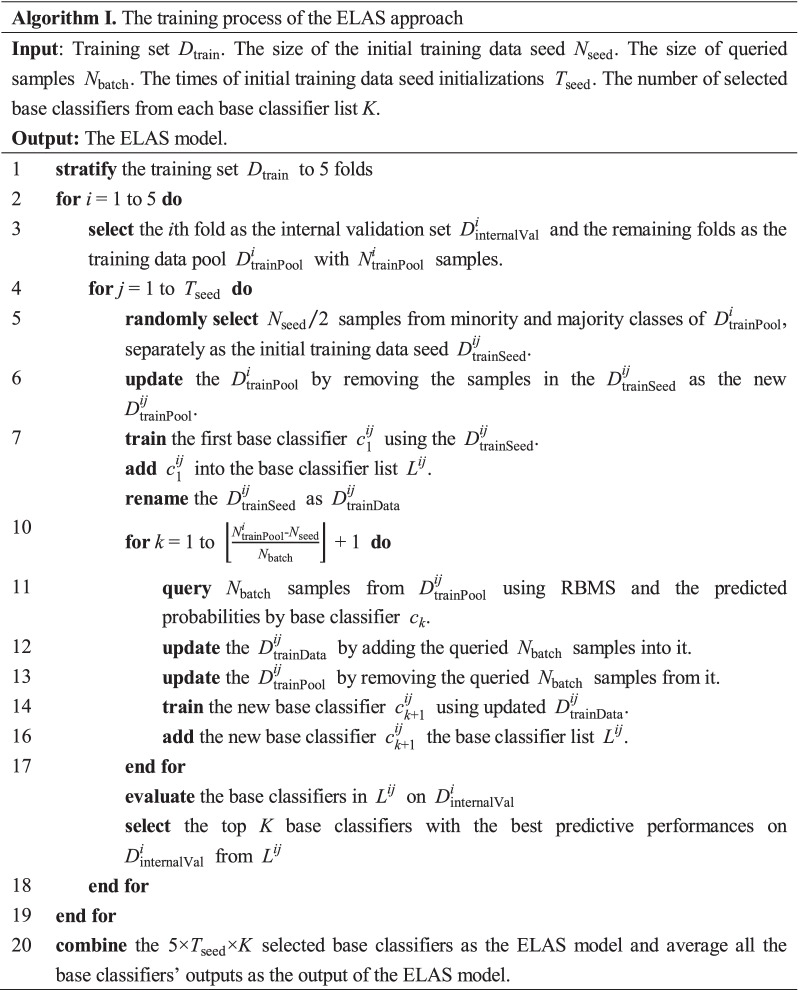


### Experimental setup

To develop the ELAS model, we selected support vector machine (SVM) [22], logistic regression with L2 regularization (L2-LR) [23], and classification and regression trees (CART) [24], to train the base classifiers. We randomly divided 80% of samples as the training set and the remaining 20% as the test set. To tune the hyper-parameters, fivefold cross-validation was employed on the training set, and a grid search strategy was applied for the base classifiers on the hyper-parameter spaces: $C \in \{ 0.1,1,10\}$ for SVM, $C \in \{ 1,10,100\}$ for L2-LR, $\max \_depth \in \{ {\text{None}},5, 10\}$ and $\min \_sample\_leaf \in \{ 1,3,5\}$ for CART. To release the problem of massive possible value sets of the hyper-parameters, we selected radial basis function kernel for SVM, Gini impurity for CART, and $N_{{{\text{seed}}}} \in \{ 50,100\}$, 10 for $N_{{{\text{batch}}}}$, 3 for $T_{{{\text{seed}}}}$, 20 for $K$. Note that we should keep the $N_{{{\text{seed}}}} /2$ no more than the sample size of minority class because we want the $D_{{{\text{trainSeed}}}}$ to be a balanced dataset. Besides, we should also keep the *K* no more than $\frac{{N_{{{\text{trainPool}}}} - N_{{{\text{seed}}}} }}{{N_{{{\text{batch}}}} }} + 1$ to ensure that the top *K* base classifiers can be selected from.

In this study, we conducted extensive experiments to explore the effectiveness of the proposed ELAS approach. First, we compared the ELAS with the base classifier algorithms, i.e., SVM, L2-LR, and CART, to explore whether the ELAS can improve the performance of prognostic prediction. And then, as the ELAS is an ensemble method, we also selected two famous ensemble methods, i.e., AdaBoost [25] and Bagging [26, 27], as the benchmarks. Moreover, we also applied two resampling methods to deal with imbalanced data, namely SMOTE [28] and TomekLinks [29], to explore which strategy is better. To evaluate the ELAS and benchmarks’ performances, we employed the area under the receiver operating characteristic curve (AUROC) and the area under the precision–recall curve (AUPRC) as the metrics. To eliminate the bias caused by the test set partition, the whole data set segmentation, model development, and evaluation process was repeated 10 times with different random seeds so that we can obtain the averaged AUROC value and AUPRC value with their standard deviations (SD) for each prognostic task. The paired student t-test was performed to determine whether the AUROC and AUPRC values of ELAS are statistically significantly different from the values of the benchmark algorithms and a *p* value less than 0.05 was considered significant.

## Results

### Data

We reviewed 1848 NSCLC patients who had undergone curative surgery from 2006 to 2015 in the Department of Thoracic Surgery II of Peking University Cancer Hospital. The collected data covered patient demographic information, preoperative exams and treatments, pathological information of the primary tumor and lymph nodes, and the pathological TNM stage. Clinicians manually recorded all the clinical data to ensure its reliability and correctness. The details of the clinical data are listed in the Additional file 1. Before model development, we preprocessed the collected clinical data. Specifically, patient samples with missing feature values were excluded from the dataset. We removed the features with variance lower than 1% to ensure that no features have almost the same value for all samples. We converted all categorical features into a one-hot encoding form and binned the continuous features into intervals.

To label the patient’s recurrence and death statuses, we used the Disease-Free-Survival (DFS) and Overall-Survival (OS) in the follow-up data. In this study, we selected three time periods, i.e., 1-year, 3-year, and 5-year, to explore the effectiveness of the proposed method to handle the different degrees of imbalance. Within each period, we labeled the patients who experienced the events (recurrence or death) as positive samples and those who did not experience any events as negative samples. Patients who lost follow-up within the period and had not experienced any events were excluded from this prognostic task. Table 1 lists the statistics of the 1-year, 3-year, and 5-year prognoses.

**Table 1:** The statistics of the 1-year, 3-year, and 5-year patient prognoses

Outcomes	Number of patients		
	1-year	3-year	5-year
Recurrence, n (%)	102 (7.6%)	296 (29.1%)	377 (51.9%)
No recurrence, n (%)	1,246 (92.4%)	720 (70.9%)	350 (48.1%)
Death, n (%)	62 (4.6%)	220 (21.8%)	307 (43.7%)
No death, n (%)	1,288 (95.4%)	787 (78.2%)	395 (56.3%)

### In comparison with the base classifier algorithms

**Fig. 2:**
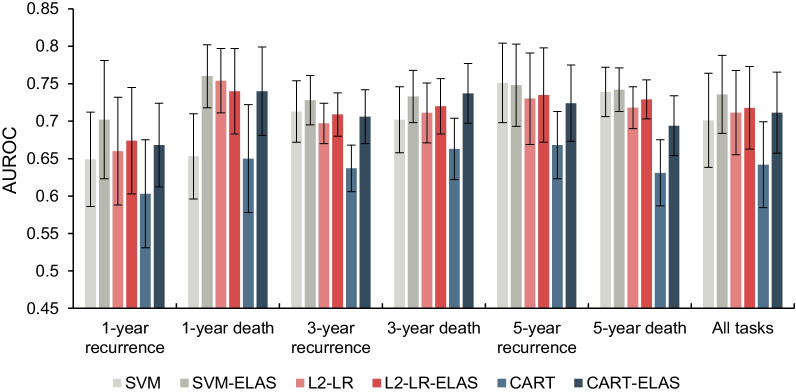
The AUROC values of the base classifier algorithms and the ELAS

**Fig. 3:**
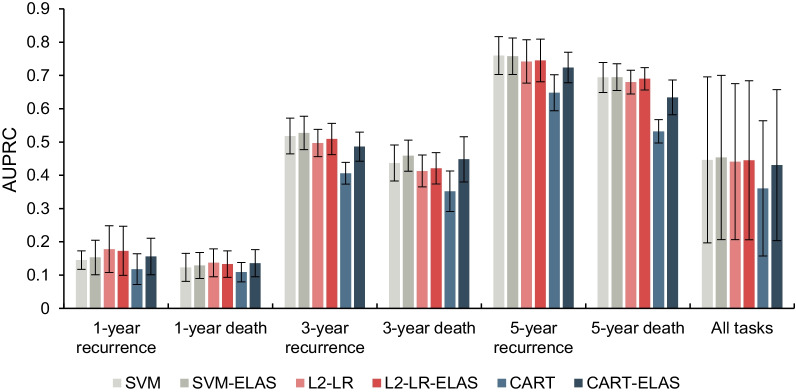
The AUPRC values of the base classifier algorithms and the ELAS

As an ensemble learning method, we first compared the ELAS with the base classifier algorithms to explore whether the base classifier algorithms can benefit from the ELAS. The AUROC and AUPRC values of the base classifier algorithms and the ELAS are illustrated in Tables 2 and 3. Figures 2 and 3 present the results in the bar graph manner. The sensitivity and specificity values are listed in the Additional file 2. We also calculated the paired student t-test to explore whether there are significant differences between the base classifier algorithms and the ELAS, and the results are listed in Table 4.

**Table 2:** The AUROC values of the base classifier algorithms and the ELAS

Task	Base classifier algorithms						ELAS					
	SVM		L2-LR		CART		SVM-ELAS		L2-LR-ELAS		CART-ELAS	
	Mean	SD	Mean	SD	Mean	SD	Mean	SD	Mean	SD	Mean	SD
1-year recurrence	0.649	0.063	0.660	0.072	0.603	0.072	**0.702**	0.079	0.674	0.071	0.668	0.056
1-year death	0.653	0.057	0.754	0.043	0.65	0.072	**0.760**	0.042	0.740	0.057	0.740	0.059
3-year recurrence	0.713	0.041	0.697	0.027	0.637	0.031	**0.728**	0.033	0.709	0.029	0.706	0.036
3-year death	0.702	0.044	0.711	0.040	0.663	0.041	0.733	0.035	0.720	0.037	**0.737**	0.040
5-year recurrence	**0.751**	0.053	0.730	0.061	0.668	0.045	0.748	0.055	0.735	0.063	0.724	0.051
5-year death	0.739	0.033	0.718	0.028	0.631	0.044	**0.742**	0.029	0.729	0.026	0.694	0.040
All tasks	0.701	0.063	0.711	0.056	0.642	0.057	**0.736**	0.052	0.718	0.055	0.711	0.054

The bold means the best results for corresponding tasks

**Table 3:** The AUPRC values of the base classifier algorithms and the ELAS

Task	Base classifier algorithms						ELAS					
	SVM		L2-LR		CART		SVM-ELAS		L2-LR-ELAS		CART-ELAS	
	Mean	SD	Mean	SD	Mean	SD	Mean	SD	Mean	SD	Mean	SD
1-year recurrence	0.145	0.028	**0.178**	0.070	0.118	0.046	0.153	0.052	0.173	0.074	0.156	0.055
1-year death	0.123	0.042	**0.137**	0.042	0.109	0.029	0.129	0.039	0.133	0.040	0.136	0.041
3-year recurrence	0.518	0.054	0.497	0.041	0.406	0.033	**0.527**	0.050	0.509	0.047	0.486	0.044
3-year death	0.437	0.054	0.413	0.048	0.352	0.061	**0.459**	0.047	0.421	0.047	0.448	0.068
5-year recurrence	**0.760**	0.057	0.742	0.065	0.648	0.054	0.758	0.055	0.745	0.064	0.724	0.046
5-year death	0.694	0.045	0.680	0.036	0.532	0.035	**0.695**	0.040	0.690	0.034	0.634	0.052
All tasks	0.446	0.250	0.441	0.234	0.361	0.203	**0.453**	0.247	0.445	0.239	0.431	0.227

The bold means the best results for corresponding tasks

**Table 4:** The paired student t-test results between the base classifier algorithms and the ELAS

Metric	Comparison	1-year tasks	3-year tasks	5-year tasks	All tasks
AUROC	SVM versus SVM-ELAS	**< 0.01**	**< 0.01**	0.411	**< 0.01**
	L2-LR versus L2-LR-ELAS	0.487	**< 0.01**	**< 0.01**	**< 0.01**
	CART versus CART-ELAS	**< 0.01**	**< 0.01**	**< 0.01**	**< 0.01**
AUPRC	SVM versus SVM-ELAS	0.165	**< 0.01**	0.378	**< 0.01**
	L2-LR versus L2-LR-ELAS	0.093	**< 0.01**	**0.011**	**0.015**
	CART versus CART-ELAS	**< 0.01**	**< 0.01**	**< 0.01**	**< 0.01**

The bold means the p-value is less than 0.05, which means the results between different models have statistically significant differences

Based on the experimental results above, we find that the ELAS achieves significant improvements compared with all base classifier algorithms under both AUROC and AUPRC metrics when combining all prognostic tasks together. Moreover, the more serious the data imbalance, the more improvements on AUROC values obtained by the ELAS, which indicates the ELAS strategy can better tackle the imbalanced problem than the base classifiers. From Fig. 3 we can notice that the AUPRC values increase in a step-like manner with the extension of the time of the prognostic prediction task, which is because the AUPRC is mainly affected by the degree of data imbalance. For 1-year prognostic prediction tasks, only CART significantly benefited from the ELAS method under AUPRC metric, but SVM and L2-LR did not gain significant improvements on AUPRC values via using ELAS. For 3-year prognostic prediction tasks, all base classifier algorithms achieve better performances when using ELAS. For 5-year prognostic prediction tasks, both L2-LR and CART have significant improvements, but SVM does not. By comparing the three ELAS models, we notice that the SVM-ELAS achieved the best overall performances with 0.736 AUROC value and 0.453 AUPRC value. So, we select the SVM-ELAS as the representative model to compare with other benchmarks in the following experiments.

### In comparison with the benchmark algorithms

Ensemble learning is one of the effective approaches to handling the imbalanced data problem [16, 17, 30]. In this study, the proposed ELAS also averages the outputs of the selected base classifiers as the final predictive result. So here, we apply two state-of-the-art ensemble learning algorithms, i.e., AdaBoost [25] and Bagging [26, 27], as the benchmarks to compare with the ELAS. AdaBoost is one popular boosting algorithm that fits a sequence of weak classifiers on repeatedly reweighted samples and follows to weighted sum the outputs of weak classifiers as the predictive results. Bagging is another ensemble strategy that randomly samples subsets of the training set without concern for performance to build base classifiers and then averages their outputs as the predictions. Unlike them, the ELAS bias towards selecting samples that are hard to distinguish to train the base classifier step by step and averages the outputs of the base classifiers with the best performances as the ensemble predictions.

Besides ensemble learning, resampling techniques are also widely used to alleviate the effect of the skewed class distribution by rebalancing the sample space for an imbalanced dataset [16, 17]. In this subsection, we also select two resampling techniques, i.e., SMOTE [28] and TomekLinks [29], as the benchmarks to compare with the proposed method. SMOTE is an over-sampling method that generates new samples from the vector between the current sample and one of its k nearest neighbors to enrich the minority class. TomekLinks is an under-sampling method that first detects if the two samples of different classes are the nearest neighbors of each other and then deletes the one in the majority class to reduce the majority class.

**Fig. 4:**
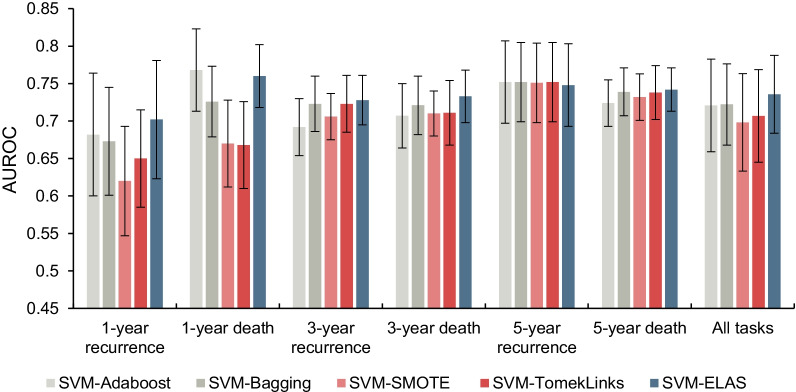
The AUROC values of the ensemble algorithms, resampling algorithms, and the ELAS

**Fig. 5:**
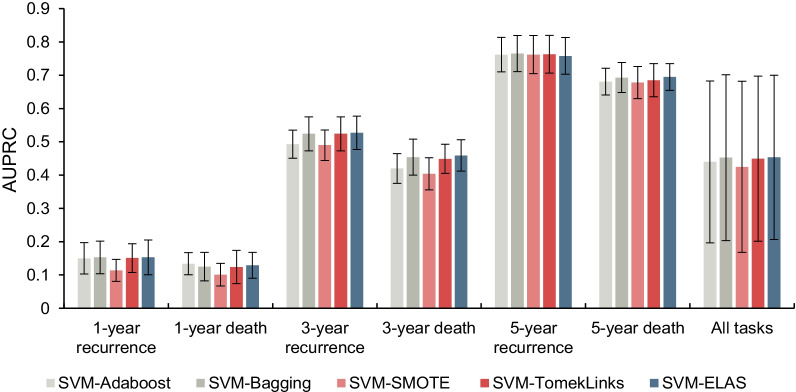
The AUPRC values of the ensemble algorithms, resampling algorithms, and the ELAS

Tables 5 and 6 show the AUROC values and AUPRC values of the benchmarks and the ELAS, respectively. Figures 4 and 5 present the experimental results more intuitively. The sensitivity and specificity values of the benchmarks and the ELAS are listed in the Additional file 3. To further prove the performance improvements of the ELAS, the paired student t-test is also conducted to compare the performances of the benchmarks and the ELAS and listed in Table 7.

**Table 5:** The AUROC values of the ensemble algorithms, resampling algorithms, and the ELAS

Task	Ensemble algorithms				Resampling algorithms				Proposed	
	SVM-AdaBoost		SVM-Bagging		SVM-SMOTE		SVM-TomekLinks		SVM-ELAS	
	Mean	SD	Mean	SD	Mean	SD	Mean	SD	Mean	SD
1-year recurrence	0.682	0.082	0.673	0.072	0.620	0.073	0.650	0.065	**0.702**	0.079
1-year death	**0.768**	0.055	0.726	0.047	0.670	0.058	0.668	0.058	0.760	0.042
3-year recurrence	0.692	0.038	0.723	0.037	0.706	0.031	0.723	0.038	**0.728**	0.033
3-year death	0.707	0.043	0.721	0.039	0.710	0.030	0.711	0.043	**0.733**	0.035
5-year recurrence	**0.752**	0.055	**0.752**	0.053	0.751	0.053	**0.752**	0.053	0.748	0.055
5-year death	0.724	0.031	0.739	0.032	0.732	0.031	0.738	0.036	**0.742**	0.029
All tasks	0.721	0.062	0.722	0.054	0.698	0.065	0.707	0.062	**0.736**	0.052

The bold means the best results for corresponding tasks

**Table 6:** The AUPRC values of the ensemble algorithms, resampling algorithms, and the ELAS

Task	Ensemble algorithms				Resampling algorithms				Proposed	
	SVM-AdaBoost		SVM-Bagging		SVM-SMOTE		SVM-TomekLinks		SVM-ELAS	
	Mean	SD	Mean	SD	Mean	SD	Mean	SD	Mean	SD
1-year recurrence	0.150	0.047	**0.153**	0.049	0.114	0.033	0.151	0.043	**0.153**	0.052
1-year death	**0.134**	0.033	0.125	0.043	0.101	0.034	0.124	0.050	0.129	0.039
3-year recurrence	0.493	0.042	0.524	0.051	0.490	0.046	0.524	0.051	**0.527**	0.050
3-year death	0.420	0.045	0.454	0.054	0.404	0.048	0.449	0.044	**0.459**	0.047
5-year recurrence	0.762	0.052	**0.765**	0.054	0.762	0.057	0.763	0.057	0.758	0.055
5-year death	0.681	0.040	0.693	0.045	0.678	0.048	0.685	0.050	**0.695**	0.040
All tasks	0.440	0.243	0.452	0.249	0.425	0.257	0.449	0.248	**0.453**	0.247

The bold means the best results for corresponding tasks

**Table 7:** The paired student t-test results between the benchmark algorithms and the ELAS

Metric	Comparison	1-year tasks	3-year tasks	5-year tasks	All tasks
AUROC	SVM-AdaBoost versus SVM-ELAS	0.231	**< 0.01**	**0.041**	**< 0.01**
	SVM-Bagging versus SVM-ELAS	**< 0.01**	**< 0.01**	0.490	**< 0.01**
	SVM-SMOTE versus SVM-ELAS	**< 0.01**	**< 0.01**	0.104	**< 0.01**
	SVM-TomekLinks versus SVM-ELAS	**< 0.01**	**< 0.01**	0.454	**< 0.01**
AUPRC	SVM-AdaBoost versus SVM-ELAS	0.428	**< 0.01**	0.096	**< 0.01**
	SVM-Bagging versus SVM-ELAS	0.396	**0.046**	0.146	0.337
	SVM-SMOTE versus SVM-ELAS	**< 0.01**	**< 0.01**	0.084	**< 0.01**
	SVM-TomekLinks versus SVM-ELAS	0.334	**0.041**	0.287	0.088

The bold means the p-value is less than 0.05, which means the results between different models have statistically significant differences

Note that the SVM-ELAS achieved the best overall performance with 0.736 AUROC value and 0.453 AUPRC value for all tasks together compared with the benchmarks. For 1-year prognostic prediction tasks, the SVM-ELAS outperformed the benchmark algorithms on AUROC values significantly except for the SVM-AdaBoost. The possible reason is that there is a good similarity between the reweighting in AdaBoost and active sampling in the ELAS. Specifically, AdaBoost gives higher weights to misclassified samples so that subsequent base classifiers can tend to classify them correctly, while ELAS actively selects the indistinguishable samples into the training data and uses these samples for all subsequent base classifier developments. So, the samples hard to classify are paid extra attention in both AdaBoost and ELAS, which may lead the similar prediction performances for the 1-year prognostic prediction tasks. Although SVM-ELAS did not outperform the SVM-AdaBoost on 1-year prognostic prediction tasks but obtained significant improvements on both metrics when combining all tasks. Compared with the resampling methods, the SVM-ELAS outperforms the benchmark models for 1-year prediction tasks except for AUPRC of SVM-TomekLinks on 1-year prediction tasks, which indicates the ELAS is a competitive strategy to handle the imbalanced data problem compared with SMOTE and TomekLinks. For 3-year prognostic tasks, the SVM-ELAS achieves significant improvements on both AUROC and AUPRC values compared with all benchmarks. But for 5-year prognostic prediction tasks, the SVM-ELAS did not show significant improvements, probably due to the data imbalance problem becoming relatively weak.

## Discussion

In this study, we proposed the ELAS to tackle the imbalanced data problem in NSCLC prognostic prediction. Our approach is generalizable for other biomedical data analyses with imbalanced prediction targets. The experimental results have demonstrated that the ELAS has robust predictive performance, especially for short-term prognostic prediction, when compared with the state-of-the-art techniques.

Although the ELAS achieves comparative performances for NSCLC prognostic prediction, there are multiple directions we would like to further work into for more meaningful discoveries.

In the current study, we just employed one kind of query strategy described in the literature [18] to select the most informative patient samples. However, exploiting multiple query criteria together shows great potential to improve the performance for classification problems [21, 31]. In the future, we can attempt to use multiple query criteria to select the representative samples from different perspectives to facilitate the development of the base classifier, e.g., using information density to take the structure of the data into account [32], combining base classifiers from different initial training data set as a committee to select the samples with the most disagreements [32].

Moreover, although we applied multiple internal validation sets for the base classifier selection to alleviate the overfitting problem, this selection strategy makes the selected base classifiers easily overfit to the corresponding internal validation set. In the future, we can attempt to sample the base classifiers using the distribution generated from the performances of base classifiers or randomly select a subset of top N classifiers to further reduce the overfitting problem.

## Conclusions

In this study, we proposed the ELAS approach to predict the prognosis for postoperative NSCLC patients. Experimental results indicate that the ELAS achieves the best overall performance with an averaged 0.736 AUROC value and 0.453 AUPRC value in comparison with the benchmark models, which indicates it can effectively alleviate the imbalanced data problem in NSCLC prognostic prediction.

## Supplementary Information

**Additional file 1.** The details of the experimental datasets.

**Additional file 2.** The sensitivity and specificity values of the base classifier algorithms and the ELAS.

**Additional file 3.** The sensitivity and specificity values of the ensemble algorithms, resampling algorithms, and the ELAS.

## Data Availability

The datasets generated and/or analyzed during the current study are not publicly available due to the hospital’s regulations, but are available from the corresponding author on reasonable request.

## Abbreviations

AUPRC: Area under the precision-recall curve
AUROC: Area under the receiver operating characteristic curve
CART: Classification and regression trees
DFS: Disease free survival
ELAS: Ensemble learning with active sampling
L2-LR: Logistic regression with L2 regularization
NSCLC: Non-small cell lung cancer
OS: Overall survival
RBMS: Ranked batch-mode sampling
ROC: Receiver operating characteristic curve
SCLC: Small-cell lung cancer
SD: Standard deviation
SMOTE: Synthetic minority over-sampling technique
SVM: Support vector machine
