# Supplementary material for: An ensemble learning with active sampling to predict the prognosis of postoperative non-small cell lung cancer patients
Source: BMC Med Inform Decis Mak. 2022 Sep 19;22:245. doi: 10.1186/s12911-022-01960-0 (PMC9487160; doi:10.1186/s12911-022-01960-0)
Supplement: Supplementary file 1 — Additional file 1. The details of the experimental datasets. [file 12911_2022_1960_MOESM1_ESM.docx]

## Additional file 1. The details of the experimental datasets.

| Characteristic | | 1-year recurrence (n=1348) | 1-year survival (n=1350) | 3-year recurrence (n=1016) | 3-year survival (n=1007) | 5-year recurrence (N=727) | 5-year survival (n=702) |
| --- | --- | --- | --- | --- | --- | --- | --- |
| **Gender, n (%)** | | | | | | | |
|  | Male | 829 (61.5) | 829 (61.4) | 628 (61.8) | 620 (61.6) | 453 (62.3) | 441 (62.8) |
|  | Female | 519 (38.5) | 521 (38.6) | 388 (38.2) | 387 (38.4) | 274 (37.7) | 261 (37.2) |
| Age, mean (SD) | | 60.19 (9.72) | 60.20 (9.72) | 60.27 (9.80) | 60.32 (9.80) | 60.34 (9.80) | 60.51 (9.80) |
| **Smoking history, n (%)** | | | | | | | |
|  | Smoker | 719 (53.3) | 719 (53.3) | 548 (53.9) | 542 (53.8) | 389 (53.5) | 381 (54.3) |
|  | Never | 629 (46.7) | 631 (46.7) | 468 (46.1) | 465 (46.2) | 338 (46.5) | 321 (45.7) |
| **Drinking history, n (%)** | | | | | | | |
|  | Drinker | 383 (28.4) | 384 (28.4) | 280 (27.6) | 275 (27.3) | 205 (28.2) | 198 (28.2) |
|  | Never | 965 (71.6) | 966 (71.6) | 736 (72.4) | 732 (72.7) | 522 (71.8) | 504 (71.8) |
| **Tumor history, n (%)** | | | | | | | |
|  | Yes | 45 (3.3) | 44 (3.3) | 33 (3.2) | 32 (3.2) | 21 (2.9) | 19 (2.7) |
|  | No | 1303 (96.7) | 1306 (96.7) | 983 (96.8) | 975 (96.8) | 706 (97.1) | 683 (97.3) |
| **Family history, n (%)** | | | | | | | |
|  | Yes | 189 (14.0) | 188 (13.9) | 141 (13.9) | 140 (13.9) | 94 (12.9) | 92 (13.1) |
|  | No | 1159 (86.0) | 1162 (86.1) | 875 (86.1) | 867 (86.1) | 633 (87.1) | 610 (86.9) |
| **Pre-treatment pathology, n (%)** | | | | | | | |
|  | Yes | 405 (30.0) | 405 (30.0) | 278 (27.4) | 276 (27.4) | 203 (27.9) | 199 (28.3) |
|  | No | 943 (70.0) | 945 (70.0) | 738 (72.6) | 731 (72.6) | 524 (72.1) | 503 (71.7) |
| **PET/CT, n (%)** | | | | | | | |
|  | Yes | 369 (27.4) | 371 (27.5) | 252 (24.8) | 248 (24.6) | 191 (26.3) | 183 (26.1) |
|  | No | 979 (72.6) | 979 (72.5) | 764 (75.2) | 759 (75.4) | 536 (73.7) | 519 (73.9) |
| **Preoperative treatment, n (%)** | | | | | | | |
|  | Yes | 178 (13.2) | 178 (13.2) | 133 (13.1) | 131 (13.0) | 90 (12.4) | 89 (12.7) |
|  | No | 1170 (86.8) | 1172 (86.8) | 883 (86.9) | 876 (87.0) | 637 (87.6) | 613 (87.3) |
| **Bronchioplasty/Angioplasty, n (%)** | | | | | | | |
|  | Yes | 160 (11.9) | 160 (11.9) | 124 (12.2) | 122 (12.1) | 90 (12.4) | 86 (12.3) |
|  | No | 1188 (88.1) | 1190 (88.1) | 892 (87.8) | 885 (87.9) | 637 (87.6) | 616 (87.7) |
| Tumor size, mean (SD) | | 3.15 (1.80) | 3.15 (1.80) | 3.20 (1.81) | 3.20 (1.81) | 3.38 (1.81) | 3.38 (1.81) |
| **Vessel carcinoma embolus, n (%)** | | | | | | | |
|  | Yes | 196 (14.5) | 197 (14.6) | 142 (14.0) | 140 (13.9) | 115 (15.8) | 109 (15.5) |
|  | No | 1152 (85.5) | 1153 (85.4) | 874 (86.0) | 867 (86.1) | 612 (84.2) | 593 (84.5) |
| **Pleural invasion, n (%)** | | | | | | | |
|  | Yes | 531 (39.4) | 533 (39.5) | 445 (43.8) | 446 (44.3) | 320 (44.0) | 309 (44.0) |
|  | No | 817 (60.6) | 817 (60.5) | 571 (56.2) | 561 (55.7) | 407 (56.0) | 393 (56.0) |
| **pT stage, n (%)** | | | | | | | |
|  | T1a | 257 (19.1) | 255 (18.9) | 172 (16.9) | 167 (16.6) | 110 (15.1) | 103 (14.7) |
|  | T1b | 232 (17.2) | 232 (17.2) | 173 (17.0) | 171 (17.0) | 122 (16.8) | 119 (17.0) |
|  | T2a | 607 (45.0) | 612 (45.3) | 470 (46.3) | 472 (46.9) | 343 (47.2) | 334 (47.6) |
|  | T2b | 95 (7.0) | 95 (7.0) | 74 (7.3) | 74 (7.3) | 58 (8.0) | 56 (8.0) |
|  | T3 | 129 (9.6) | 128 (9.5) | 101 (9.9) | 97 (9.6) | 75 (10.3) | 71 (10.1) |
|  | T4 | 28 (2.1) | 28 (2.1) | 26 (2.6) | 26 (2.6) | 19 (2.6) | 19 (2.7) |
| **pN stage, n (%)** | | | | | | | |
|  | N0 | 859 (63.7) | 861 (63.8) | 626 (61.6) | 622 (61.8) | 408 (56.1) | 398 (56.7) |
|  | N1 | 228 (16.9) | 228 (16.9) | 178 (17.5) | 174 (17.3) | 138 (19.0) | 130 (18.5) |
|  | N2 | 261 (19.4) | 261 (19.3) | 212 (20.9) | 211 (21.0) | 181 (24.9) | 174 (24.8) |
| **Surgery type, n (%)** | | | | | | | |
|  | Pneumonectomy | 41 (3.0) | 41 (3.0) | 34 (3.3) | 33 (3.3) | 25 (3.4) | 25 (3.6) |
|  | Wedge resection | 47 (3.5) | 47 (3.5) | 41 (4.0) | 40 (4.0) | 26 (3.6) | 25 (3.6) |
|  | Lobectomy | 1167 (86.6) | 1169 (86.6) | 863 (84.9) | 860 (85.4) | 624 (85.8) | 605 (86.2) |
|  | Bilobectomy | 85 (6.3) | 85 (6.3) | 72 (7.1) | 69 (6.9) | 48 (6.6) | 44 (6.3) |
|  | Segmentectomy | 5 (0.4) | 5 (0.4) | 4 (0.4) | 4 (0.4) | 2 (0.3) | 2 (0.3) |
|  | Others | 3 (0.2) | 3 (0.2) | 2 (0.2) | 1 (0.1) | 2 (0.3) | 1 (0.1) |
| **Tumor location, n (%)** | | | | | | | |
|  | Right upper lobe | 429 (31.8) | 429 (31.8) | 324 (31.9) | 319 (31.7) | 234 (32.2) | 225 (32.1) |
|  | Right middle lobe | 83 (6.2) | 83 (6.1) | 65 (6.4) | 63 (6.3) | 46 (6.3) | 43 (6.1) |
|  | Right lower lobe | 273 (20.3) | 273 (20.2) | 209 (20.6) | 209 (20.8) | 155 (21.3) | 149 (21.2) |
|  | Left upper lobe | 303 (22.5) | 303 (22.4) | 224 (22.0) | 221 (21.9) | 153 (21.0) | 149 (21.2) |
|  | Left lower lobe | 222 (16.5) | 224 (16.6) | 169 (16.6) | 170 (16.9) | 121 (16.6) | 118 (16.8) |
|  | Others | 38 (2.8) | 38 (2.8) | 25 (2.5) | 25 (2.5) | 18 (2.5) | 18 (2.6) |
| **Histologic type, n (%)** | | | | | | | |
|  | Large cell carcinoma | 26 (1.9) | 26 (1.9) | 24 (2.4) | 24 (2.4) | 17 (2.3) | 17 (2.4) |
|  | Adenocarcinoma | 848 (62.9) | 849 (62.9) | 643 (63.3) | 639 (63.5) | 459 (63.1) | 439 (62.5) |
|  | Squamous cell carcinoma | 427 (31.7) | 428 (31.7) | 310 (30.5) | 306 (30.4) | 225 (30.9) | 222 (31.6) |
|  | Adenosquamous carcinoma | 18 (1.3) | 18 (1.3) | 16 (1.6) | 15 (1.5) | 12 (1.7) | 11 (1.6) |
|  | Others | 29 (2.2) | 29 (2.1) | 23 (2.3) | 23 (2.3) | 14 (1.9) | 13 (1.9) |
| **Grade, n (%)** | | | | | | | |
|  | Well differentiated | 239 (17.7) | 240 (17.8) | 143 (14.1) | 144 (14.3) | 94 (12.9) | 91 (13.0) |
|  | Moderately differentiated | 578 (42.9) | 578 (42.8) | 448 (44.1) | 441 (43.8) | 304 (41.8) | 287 (40.9) |
|  | Poorly differentiated | 529 (39.2) | 530 (39.3) | 423 (41.6) | 420 (41.7) | 327 (45.0) | 322 (45.9) |
|  | Others | 2 (0.1) | 2 (0.1) | 2 (0.2) | 2 (0.2) | 2 (0.3) | 2 (0.3) |
| **Lymph node metastasis, n/n** | | | | | | | |
|  | 1L Positive/1L Collected | 0/6 | 0/6 | 0/5 | 0/5 | 0/1 | 0/0 |
|  | 1R Positive/1R Collected | 7/91 | 7/91 | 7/82 | 7/82 | 7/67 | 7/66 |
|  | 2L Positive/2L Collected | 1/5 | 1/5 | 1/3 | 1/3 | 1/3 | 1/3 |
|  | 2R Positive/2R Collected | 31/343 | 31/344 | 27/262 | 27/259 | 24/202 | 24/197 |
|  | 2R+4R Positive/2R+4R Collected | 51/335 | 51/335 | 39/207 | 37/201 | 31/117 | 29/101 |
|  | 3a Positive/3a Collected | 20/230 | 20/230 | 17/200 | 17/199 | 16/132 | 16/126 |
|  | 3p Positive/3p Collected | 4/97 | 4/98 | 3/81 | 3/82 | 3/58 | 3/55 |
|  | 4L Positive/4L Collected | 21/209 | 21/209 | 16/163 | 16/162 | 12/117 | 12/116 |
|  | 4R Positive/4R Collected | 70/465 | 70/464 | 65/388 | 65/385 | 59/306 | 59/299 |
|  | 5 Positive/5 Collected | 40/408 | 40/409 | 32/293 | 32/291 | 28/213 | 27/210 |
|  | 6 Positive/6 Collected | 45/429 | 44/432 | 32/309 | 31/308 | 24/209 | 22/203 |
|  | 7 Positive/7 Collected | 135/1226 | 136/1229 | 112/909 | 114/901 | 101/659 | 99/636 |
|  | 8 Positive/8 Collected | 10/220 | 10/221 | 8/181 | 8/178 | 8/146 | 8/145 |
|  | 9 Positive/9 Collected | 20/837 | 20/839 | 18/657 | 18/654 | 16/467 | 16/450 |
|  | 10 Positive/10 Collected | 142/1078 | 141/1079 | 118/822 | 117/816 | 105/602 | 103/584 |
|  | 11 Positive/11 Collected | 72/798 | 72/799 | 58/592 | 58/588 | 51/435 | 49/417 |
|  | 12 Positive/12 Collected | 234/1082 | 235/1084 | 188/805 | 185/799 | 153/571 | 148/554 |
|  | 13 Positive/13 Collected | 139/671 | 140/671 | 103/437 | 101/432 | 78/278 | 72/264 |
|  | 14 Positive/14 Collected | 50/285 | 50/287 | 40/222 | 40/224 | 32/163 | 31/160 |
