# Supplementary material for: An ensemble learning with active sampling to predict the prognosis of postoperative non-small cell lung cancer patients
Source: BMC Med Inform Decis Mak. 2022 Sep 19;22:245. doi: 10.1186/s12911-022-01960-0 (PMC9487160; doi:10.1186/s12911-022-01960-0)
Supplement: Supplementary file 2 — Additional file 2. The sensitivity and specificity values of the base classifier algorithms and the ELAS. [file 12911_2022_1960_MOESM2_ESM.docx]

## Additional file 2. The sensitivity and specificity values of the base classifier algorithms and the ELAS.

| **Task** | | **Base classifier algorithms** | | | | | | **ELAS** | | | | | |
| --- | --- | --- | --- | --- | --- | --- | --- | --- | --- | --- | --- | --- | --- |
|  |  | **SVM** | | **L2-LR** | | **CART** | | **SVM-ELAS** | | **L2-LR-ELAS** | | **CART-ELAS** | |
|  |  | **Mean** | **SD** | **Mean** | **SD** | **Mean** | **SD** | **Mean** | **SD** | **Mean** | **SD** | **Mean** | **SD** |
| 1-year recurrence | sen | 0.642 | 0.094 | 0.667 | 0.111 | 0.730 | 0.137 | **0.698** | 0.064 | 0.640 | 0.070 | 0.743 | 0.107 |
|  | spe | 0.649 | 0.076 | 0.633 | 0.101 | 0.550 | 0.056 | **0.656** | 0.095 | 0.671 | 0.084 | 0.588 | 0.109 |
| 1-year death | sen | 0.738 | 0.136 | 0.778 | 0.073 | 0.600 | 0.135 | **0.772** | 0.136 | 0.778 | 0.129 | 0.721 | 0.076 |
|  | spe | 0.594 | 0.121 | 0.679 | 0.033 | 0.696 | 0.124 | **0.717** | 0.084 | 0.684 | 0.088 | 0.719 | 0.079 |
| 3-year recurrence | sen | 0.685 | 0.072 | 0.633 | 0.049 | 0.645 | 0.064 | **0.685** | 0.069 | 0.661 | 0.061 | 0.699 | 0.048 |
|  | spe | 0.698 | 0.046 | 0.701 | 0.048 | 0.620 | 0.096 | **0.706** | 0.043 | 0.696 | 0.035 | 0.661 | 0.060 |
| 3-year death | sen | 0.664 | 0.083 | 0.688 | 0.077 | 0.651 | 0.096 | 0.693 | 0.049 | 0.683 | 0.057 | **0.678** | 0.049 |
|  | spe | 0.693 | 0.029 | 0.682 | 0.059 | 0.641 | 0.077 | 0.710 | 0.056 | 0.708 | 0.055 | **0.734** | 0.055 |
| 5-year recurrence | sen | 0.709 | 0.069 | 0.726 | 0.082 | 0.615 | 0.063 | **0.690** | 0.081 | 0.700 | 0.067 | 0.680 | 0.070 |
|  | spe | 0.694 | 0.052 | 0.665 | 0.057 | 0.694 | 0.090 | **0.715** | 0.051 | 0.696 | 0.074 | 0.699 | 0.081 |
| 5-year death | sen | 0.699 | 0.057 | 0.674 | 0.050 | 0.617 | 0.087 | **0.712** | 0.059 | 0.689 | 0.063 | 0.689 | 0.055 |
|  | spe | 0.710 | 0.072 | 0.685 | 0.053 | 0.644 | 0.088 | **0.700** | 0.079 | 0.688 | 0.051 | 0.649 | 0.071 |
| All tasks | sen | 0.689 | 0.095 | 0.694 | 0.091 | 0.643 | 0.111 | **0.708** | 0.087 | 0.692 | 0.090 | 0.702 | 0.075 |
|  | spe | 0.673 | 0.083 | 0.674 | 0.066 | 0.641 | 0.104 | **0.701** | 0.074 | 0.690 | 0.069 | 0.675 | 0.093 |
