# Supplementary material for: An ensemble learning with active sampling to predict the prognosis of postoperative non-small cell lung cancer patients
Source: BMC Med Inform Decis Mak. 2022 Sep 19;22:245. doi: 10.1186/s12911-022-01960-0 (PMC9487160; doi:10.1186/s12911-022-01960-0)
Supplement: Supplementary file 3 — Additional file 3. The sensitivity and specificity values of the ensemble algorithms, resampling algorithms, and the ELAS. [file 12911_2022_1960_MOESM3_ESM.docx]

## Additional file 3. The sensitivity and specificity values of the ensemble algorithms, resampling algorithms, and the ELAS.

| **Task** | | **Ensemble algorithms** | | | | | **Resampling algorithms** | | | | | **Proposed** | |
| --- | --- | --- | --- | --- | --- | --- | --- | --- | --- | --- | --- | --- | --- |
|  |  | **SVM-AdaBoost** | | **SVM-Bagging** | | **SVM-SMOTE** | | | **SVM-TomekLinks** | | **SVM-Bagging** | | |
|  |  | **Mean** | **SD** | **Mean** | **SD** | **Mean** | | **SD** | **Mean** | **SD** | **Mean** | | **SD** |
| 1-year recurrence | sen | 0.742 | 0.107 | 0.653 | 0.082 | 0.634 | | 0.059 | 0.628 | 0.122 | **0.698** | | 0.064 |
|  | spe | 0.607 | 0.084 | 0.665 | 0.105 | 0.599 | | 0.097 | 0.667 | 0.087 | **0.656** | | 0.095 |
| 1-year death | sen | 0.758 | 0.099 | 0.787 | 0.105 | 0.740 | | 0.111 | 0.704 | 0.116 | **0.772** | | 0.136 |
|  | spe | 0.730 | 0.060 | 0.626 | 0.063 | 0.603 | | 0.085 | 0.624 | 0.097 | **0.717** | | 0.084 |
| 3-year recurrence | sen | 0.651 | 0.067 | 0.678 | 0.074 | 0.672 | | 0.045 | **0.695** | 0.072 | 0.685 | | 0.069 |
|  | spe | 0.679 | 0.057 | 0.713 | 0.046 | 0.680 | | 0.033 | **0.706** | 0.057 | 0.706 | | 0.043 |
| 3-year death | sen | 0.652 | 0.083 | 0.690 | 0.065 | 0.662 | | 0.067 | 0.687 | 0.086 | **0.693** | | 0.049 |
|  | spe | 0.705 | 0.055 | 0.706 | 0.051 | 0.701 | | 0.055 | 0.703 | 0.053 | **0.710** | | 0.056 |
| 5-year recurrence | sen | **0.724** | 0.059 | 0.712 | 0.058 | 0.704 | | 0.059 | 0.694 | 0.072 | 0.690 | | 0.081 |
|  | spe | **0.691** | 0.055 | 0.690 | 0.048 | 0.705 | | 0.057 | 0.713 | 0.049 | 0.715 | | 0.051 |
| 5-year death | sen | 0.691 | 0.062 | 0.696 | 0.062 | 0.685 | | 0.069 | 0.706 | 0.054 | **0.712** | | 0.059 |
|  | spe | 0.684 | 0.082 | 0.700 | 0.059 | 0.703 | | 0.050 | 0.698 | 0.078 | **0.700** | | 0.079 |
| All tasks | sen | 0.703 | 0.092 | 0.703 | 0.087 | 0.683 | | 0.079 | 0.686 | 0.095 | **0.708** | | 0.087 |
|  | spe | 0.683 | 0.077 | 0.683 | 0.072 | 0.665 | | 0.082 | 0.685 | 0.079 | **0.701** | | 0.074 |
